# Calorie-Counting Apps for Monitoring and Managing Calorie Intake in Adults Living With Weight-Related Chronic Diseases: Decade-Long Scoping Review (2013-2024)

**DOI:** 10.2196/64139

**Published:** 2026-04-01

**Authors:** Kaylee Dugas, Marie-Andrée Giroux, Abdelatif Guerroudj, Jazna Leger, Asal Rouhafzay, Ghazal Rouhafzay, Jalila Jbilou

**Affiliations:** 1 Centre de Formation Médicale du Nouveau-Brunswick Moncton, NB Canada; 2 Université de Sherbrooke Sherbrooke, QC Canada; 3 School of Psychology Université de Moncton Moncton, NB Canada; 4 Department of Computer Science Université de Moncton Moncton, NB Canada

**Keywords:** calorie counting, clinical feasibility, mHealth, obesity management, weight-related chronic diseases

## Abstract

**Background:**

Overweight and obesity, as defined by the World Health Organization, correspond to BMI values of 25-29.9 kg/m² for overweight and ≥30 kg/m² for obesity. Both conditions remain major public health challenges worldwide due to their strong link with type 2 diabetes, cardiovascular disease, and hypertension, which place a heavy clinical and economic burden on health care systems. In Canada, obesity rates are notably high, with vulnerable populations disproportionately affected due to socioeconomic barriers, limited access to preventive care, and higher comorbidity rates. Calorie-counting Mobile health (mHealth) apps support dietary self-monitoring and weight control; however, varied designs and evidence complicate assessment of feasibility and effectiveness.

**Objective:**

This study aimed to systematically evaluate the structure and content of 46 calorie-counting apps, identify factors related to their acceptability and feasibility among adults living with obesity or weight-related chronic diseases, and formulate evidence-based recommendations for app developers, clinicians, and researchers.

**Methods:**

We conducted a scoping review of papers on calorie-counting apps published between January 2013 and March 2024. We identified 771 records and applied the PRISMA-ScR (Preferred Reporting Items for Systematic reviews and Meta-Analyses extension for Scoping Reviews) eligibility criteria. Data on app functions, features, user engagement, and acceptability and feasibility among adults with overweight or related chronic conditions were synthesized to generate practical recommendations for designing and clinically implementing calorie-counting apps.

**Results:**

A total of 68 studies met the inclusion criteria. Randomized controlled trials (23/68, 34%) and cohort studies (16/68, 24%) were the most common designs. Most studies targeted adults with overweight or obesity (53/68, 78%), while diabetes and hypertension were less frequently represented. In total, 46 distinct calorie-counting apps were identified, with MyFitnessPal and Lose It! being the most frequently studied. Nearly all apps (45/46, 98%) offered calorie logging, often through manual entry supported by food databases, and about half included goal-setting features. The most cited acceptability factors were personalization, automation, user-friendly design, and data sharing with health care professionals; barriers included technical issues, limited food databases, and manual entry. Adherence declined over time. For example, self-monitoring with MyFitnessPal decreased from 5.4 to 1.4 days per week from weeks 4 to 12, while use of Lose It! dropped to 4 days per week by the end of 12 weeks. Twelve recommendations were developed to enhance the feasibility and acceptability of calorie-counting apps for people living with weight-related chronic diseases.

**Conclusions:**

Calorie-counting apps hold potential as tools for supporting individuals living with obesity and weight-related chronic diseases. To improve clinical usability, app developers should enhance engagement via personalization and automation, ensuring food database comprehensiveness, and minimizing tracking effort. Further research should validate effectiveness and strategies for sustaining adherence, thereby informing development of user-friendly mHealth interventions.

## Introduction

One of the major challenges that health care systems around the world are currently facing is the prevalence of weight-related chronic diseases [1]. According to the World Health Organization, an estimated 2.5 billion adults aged 18 years and older were living with overweight, and 890 million were living with obesity in 2022. This corresponds to 43% and 16% of the adult population being overweight or obese, respectively. In Canada, the prevalence of obesity is even higher, with just over 26% of Canadians having a BMI of 30 or above in 2016 [2]. These Canadian statistics vary greatly within the country, with Atlantic provinces showing the highest figures. Notably, New Brunswick currently has the highest rates of obesity at 34.1%. While there can be many contributing factors, low income (42% of households earning less than CAD $60,000 [US $43,842] a year), low education rates (40% of the population having not completed postsecondary studies), and poor health behaviors account for the most significantly lacking determinants of health [3]. These figures are worrisome not only because of their drastic increase within the past 30 years [2], but also due to the well-established link between obesity and several health conditions, notably type 2 diabetes, cardiovascular diseases, and hypertension [4-6]. These weight-related chronic illnesses all lead to significant costs for society [7].

Tackling the epidemic of obesity and weight-related chronic diseases can be complex, especially when considering the impact of socioeconomic factors linked to obesity [2]. In general, the therapeutic approach includes interventions aiming to create a negative calorie balance, which can notably be achieved by dietary self-monitoring [8]. Current dietary self-monitoring methods include the traditional paper food diaries, wearable devices, websites, personal digital assistants, and mobile phone apps [9]. With the current smartphone ownership rates at 90% in certain countries, such as the United States [10], and the COVID-19 pandemic, which encouraged the use of telemedicine [11], mobile health (mHealth) apps in the form of calorie-counting apps show a promising field for calorie intake control. Such apps represent novel approaches that should be included in dieticians’ and health care professionals’ toolkit to help patients manage weight and weight-related chronic illnesses [12].

The increase in popularity of calorie-counting apps has prompted many studies to attempt to establish their efficacy in achieving weight loss [13,14]. A systematic review and meta-analysis of 12 randomized controlled trials demonstrated that mobile phone apps help achieve significant weight loss and BMI reduction in comparison with control groups, thus demonstrating the potential for mobile phone apps in weight management [15]. Calorie-counting apps are very heterogeneous in terms of functionalities, features, and objectives, making it difficult to compare them and provide evidence [16]. This, combined with the fact that calorie-counting apps often see a drop in adherence after only 3 to 5 weeks of use in most cases [17], contributes to difficulties with establishing clinical feasibility. Therefore, before implementing these apps as weight management options in a clinical setting, there is a clear need for studies to analyze key design elements and to identify the factors increasing the acceptability and feasibility of these calorie-counting apps. Some studies have already begun tackling this topic, but have either not evaluated calorie-counting apps specifically or have only studied such questions in healthy adult populations, and not in patients living with weight-related chronic illnesses.

As illustrated, it is urgent to establish the state of knowledge on existing apps and thereby identify knowledge gaps for future direction of research on calorie-counting apps in an adult population living with obesity and weight-related chronic disease. Accordingly, this paper explores the structure and content of calorie-counting apps and examines the factors influencing their acceptability and feasibility among adults living with obesity or weight-related chronic disease. The findings also inform evidence-based recommendations for app developers, clinicians, and researchers.

## Methods

### Overview

Scoping reviews are conducted to map existing theoretical and empirical research on a given topic. The main aims of a scoping review are to identify gaps in knowledge and theories and propose directions for future research. Thus, considering the aims of our study, a scoping review is the most appropriate and structured approach to map the literature. This scoping review was guided by the methodological guidance for scoping reviews as initially proposed by Arksey and O’Malley [18] and updated by Levac et al [19]. The manuscript was drafted according to the PRISMA-ScR (Preferred Reporting Items for Systematic Reviews and Meta-Analyses extension for Scoping Reviews) checklist [20]. The study protocol was not registered and was not published.

### Search Strategy

The search strategy was built through team discussion. The research team consisted of 4 medical students and 1 postdoctoral fellow in data science, working under the supervision of a public health physician holding a PhD in community health, with recognized expertise in health care management and implementation science. Literature search was conducted on May 17, 2024, in PubMed for papers published from January 2013 to March 2024. A period of 10 years was considered sufficient, given the large number of studies published and the rapid advancement of knowledge on the topic. Our search strategy included the following five main concepts: (1) calorie-counting apps, (2) weight-related chronic diseases, (3) calorie intake or weight, (4) acceptability and feasibility, and (5) structure, features, and functionalities. For each concept, we used a list of keywords presented in Textbox 1. These terms were either formatted as free-text words or as controlled vocabulary (Medical Subject Headings; MeSH) in PubMed and IEEE. Asterisks were used at the root of certain words when relevant to extend the scope of the Boolean search. The search was restricted to titles and abstracts. Selected papers were handled by the bibliographic reference manager, Zotero [21], then exported in Microsoft Excel form for paper screening and data extraction.

**List of keywords used in PubMed and IEEE.**

**Calorie-counting apps**

- App to count calories
- Apps for tracking calories
- Apps for tracking food
- Calorie counting app
- Calorie smart phone app
- Calorie tracking app
- Food logging app
- Diet app
- Diet tracker
- Diet tracking app
- Diet-tracking app
- Dietary mobile app
- Food imaging app
- Food monitoring app
- Food intake monitoring app
- Food intake tracking app
- Food picture app
- Food recognition
- Food recognition app
- Food balance estimation
- Food scan
- Food-scan
- Foodscan
- Nutrition app
- Nutrition tracking app
- Weight app
- Weight management app

**Weight-related chronic diseases**

- Cardiovascular disease
- Cardiovascular Diseases (MeSH)
- Chronic disease
- Chronic Disease (MeSH)
- Chronic health condition
- Chronic illness
- Diabetes
- Type 2 diabetes
- Diabetic patients
- Diabetes Mellitus (MeSH)
- Heart disease
- Hypertension
- Hypertension (MeSH)
- Long-term health condition
- Metabolic Syndrome (MeSH)
- Obese
- Obesity
- Obesity (MeSH)
- Overweight (MeSH)

**Calorie intake or weight**

- Body Mass Index (MeSH)
- Body Weight Changes (MeSH)
- Diet control
- Diet, Reducing (MeSH)
- Dieting
- Weight control
- Weight Gain (MeSH)
- Weight loss
- Weight Loss (MeSH)
- Weight management
- Weight monitor
- Weight reduction

**Acceptability and feasibility**

- Acceptability
- Feasibility
- System Usability Scale
- Usability
- Usefulness

**Structure, features, and functionalities**

- Content
- Design
- Feature
- Function
- Structure

To identify factors related to acceptability, each publication was independently reviewed by 4 authors to extract narrative descriptions of users’ and health care professionals’ perceptions of the apps, including aspects such as usability, usefulness, satisfaction, and perceived barriers. Discrepancies between reviewers were resolved through discussion with the principal investigator.

### Study Selection

The initial search strategy led to 771 records. After removing duplicates, 639 studies were retrieved for screening. We applied a preset list of inclusion and exclusion criteria to identify relevant papers (Table 1). A total of 4 team members discussed the screening strategy and completed a calibration using 4.5% (29/639) papers to ensure interrater reliability. Disagreements regarding inclusion were resolved through team discussion, with the principal investigator serving as referee. Following the PRISMA flowchart (see Results section), we retained 68 papers for data extraction. The principal investigator then reviewed the full list of papers for data synthesis.

**Table 1:** Criteria for inclusion and exclusion of papers.

Criteria	Inclusion	Exclusion
Population	Participants living with weight-related chronic disease (including overweight or obesity, prediabetes or diabetes, prehypertension or hypertension, cardiovascular disease, and metabolic syndrome) Adult population (aged 18 years and older) Human participants	Papers targeting participants with mental illnesses or eating disorders Papers targeting pediatric participants exclusively
Intervention	Use of a calorie-counting app English or French app	Use of health apps that do not include calorie-counting features (eg, exercise tracker, heart rate tracker) Intervention limited to using text messages only Use of app for data collection only (eg, phone-based questionnaires)
Comparison	Not restricted	—^a^
Outcome	Not restricted	—
Study design	Quantitative design Qualitative design	Systematic review, narrative review, scoping review, and case reports
Type of documents	Peer-reviewed papers	Conference abstracts, conference proceedings, book chapters, letters to editors, opinion papers, governmental reports, theses, policy reports, and papers unavailable through institutional access
Dates	Published between January 1, 2013, and March 31, 2024	—
Language	Full text available in English or French	Abstract in English or French, but full text in another language All other languages

^a^Not applicable.

Although specific keywords related to the outcome of interest in our study were used in the search strategy, the outcomes did not represent the inclusion criteria that needed to be present in papers. In fact, the final set of papers needed to include papers describing the app structure and functionalities. It was not an inclusion criterion whether these papers aimed to address the effects of calorie-counting apps on weight management, monitoring, and control, or on healthy eating, or to evaluate the acceptability and feasibility of these apps.

### Data Extraction

Data were extracted using a standardized form developed in Excel and approved by all authors. The variables extracted are described in Table S1 in Multimedia Appendix 1. We systematically collected information on the following:

- Study characteristics: geographic location, research protocol, and type of weight-related chronic disease in the study population.
- App characteristics: names and objectives of the calorie-counting apps, logging-in features and functionalities, calorie-counting functionalities, goal-setting functionalities, user interaction features, and aspects related to validity, accuracy, and reliability.
- Determinants of acceptability: user and health care professional perspectives, including information on user-friendliness, usability, usefulness, satisfaction, and barriers limiting acceptability.
- Determinants of feasibility: user and health care professional perspectives, including the lack of certain functionalities, accuracy of calorie-counting features, socioeconomic barriers, and data privacy concerns.

Data on adherence were also extracted inductively during the data extraction process, as adherence emerged as a recurring theme across the included studies.

To ensure intercoder reliability, daily team meetings were organized, and a calibration exercise consisting of data extraction from 5 papers (7%) was performed. The principal investigator acted as referee in cases of disagreement.

Notably, information on intervention duration was extracted when available from the included studies; however, this variable was not reported in all sources.

### Data Analysis and Synthesis

After extracting information from the included papers, we performed descriptive statistics (eg, count and proportions) to present an overview of the main characteristics of the retrieved studies (including geographic location, type of design, and weight-related chronic diseases). The results were summarized using a qualitative approach, built upon thematic content analysis, to synthesize, organize, and conceptualize findings. Whenever a dimension or an aspect could not be sorted into an existing category of concepts, we invited a meeting to discuss the possibilities of classification or the creation of a new conceptual category. The final conceptual categorization first included features and functionalities of logging in, calorie-counting, goal setting, app interactions with users, other key features, and validity. It then included factors contributing to acceptability, feasibility, and adherence (see full list in results).

## Results

### Description of Included Papers

**Figure 1:**
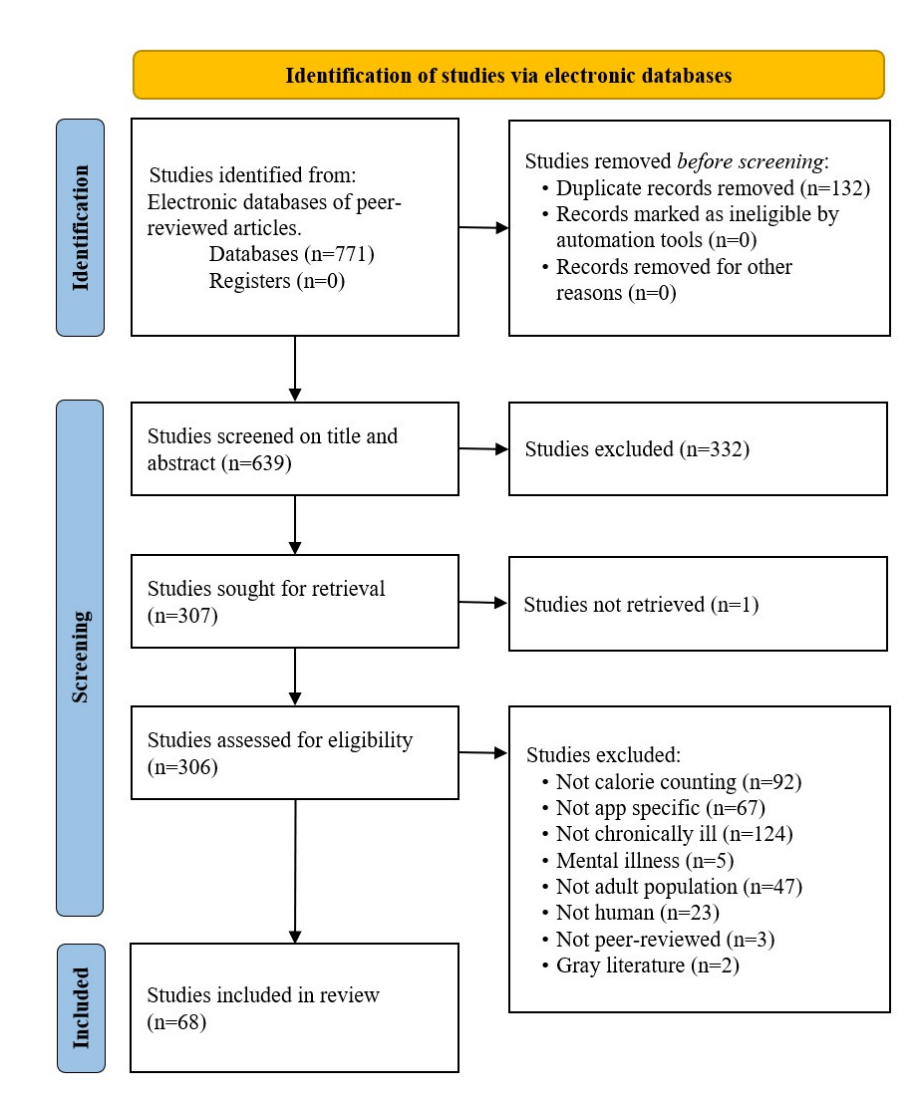
PRISMA (Preferred Reporting Items for Systematic Reviews and Meta-Analyses) flow diagram. Note that excluded papers can fall under multiple exclusion criteria.

**Figure 2:**
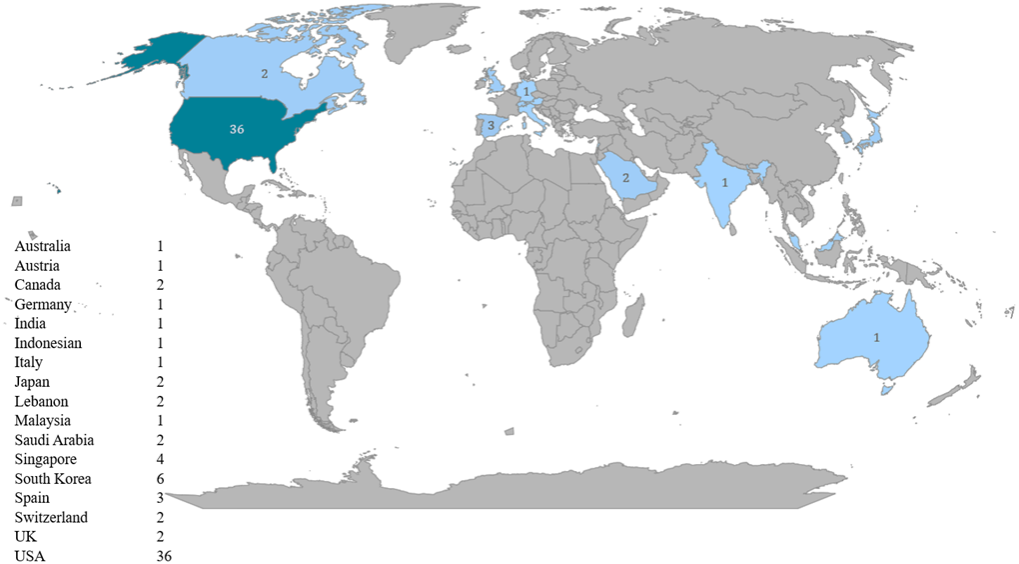
Distribution of included articles by geographic location.

Of the 68 retrieved papers [13-17,22-85], (Figure 1), most studies were conducted in the United States (36/68, 52.9%), followed by South Korea (6/68, 8.8%) and Singapore (4/68, 5.9%; Figure 2).

Randomized controlled trial (23/68, 34%) and cohort study (16/68, 24%) represented the main research designs used, followed by cross-sectional study (9/68, 13%), mixed methods design (5/68, 7%), qualitative study (5/68, 7%), and quasi-experimental study (10/68, 15%; Table 2).

**Table 2:** Distribution of included papers by study design.

Study design	Papers, n (%)
Randomized controlled trial	23 (34)
Cohort study	16 (24)
Cross-sectional study	9 (13)
Mixed method design (qualitative-quantitative)	5 (7)
Qualitative study	5 (7)
Quasi-experimental study	10 (15)

Among the 68 retrieved papers [13-17,22-85], 53 (78%) studies included participants living with obesity [13-17,22-31,36,37,40,42-44,49-53]. Participants with prediabetes or diabetes (type I or II) and prehypertension or hypertension were included in 18 of 68 (26.5%) [27-30,36,37,40,42-44,50,51,53,55,58,59,75,78] and 10 of 68 (14.7%) papers [28,30,40,43,52,64-66,69,81], respectively. Multimorbidity, or the presence of more than one weight-related chronic disease within a study population, was identified in 18 of 68 (26.5%) papers [22,27-30,36,37,40,42-44,50-53,59,69,81]. For the other weight-related chronic diseases included in this scoping review, cardiovascular disease was only found in 1 (1.5%) paper [81], and metabolic syndrome was not identified in any paper.

Out of the papers reviewed, only 19 of 68 (27.9%) provided detailed or brief descriptions of the theoretical or conceptual foundations of calorie-counting apps [22,26,29,32-36,39,41,45-48,50,54-56,72]. Among these, the social cognitive theory appeared in 8 of 68 (11.8%) studies [22,27-29,37,43,72,82]. Self-management education and chronic disease self-management frameworks were each used in 4 of 68 (5.9%) studies [25,27,51,82]. Additionally, the self-regulation theory and behavioral theory were each referenced in 3 of 68 (4.4%) papers. The goal setting theory was mentioned in 2 of 68 (2.9%) papers [68,69], while the information–motivation–behavioral skills model and behavioral economics theory were each used in a single paper.

**Figure 3:**
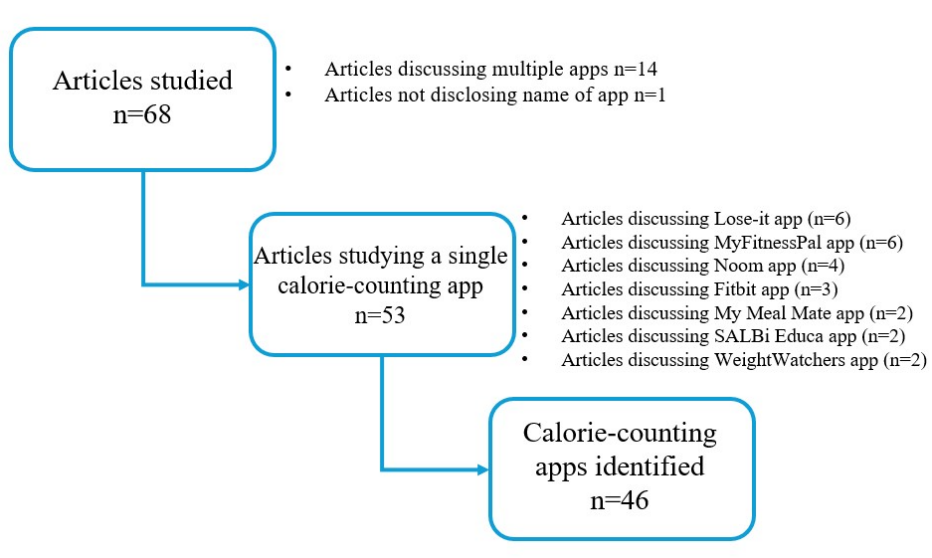
Flowchart illustrating process of identifying calorie counting apps through selected articles.

Among the 68 papers identified through our search, 14 (20.6%) examined more than one calorie-counting app [23,24,26,28,49,50,60,65,66,73,77,78,80,85]. Within this subset, 7 papers did not provide a detailed evaluation or noted commonalities and differences among the apps [23,24,26,50,65,73,80], while the other 7 conducted detailed comparisons and evaluations [28,49,60,66,77,78,85]. One paper (1.5%) did not disclose the name of the app studied [69]. The remaining 53 papers (78%) focused on evaluating or discussing a single app [13-17,22,25,27,29-48,51-59,61-64,67-72,74-76,79,81-84]. In sum of all papers, MyFitnessPal was the most frequently studied app (20/68, 29.4%) [15,16,23,24,28,46,48,49,52,54,57,60,63,67,76,79-81,84,85], followed by Lose It! (14/68, 20.6%) [15,16,23,24,31,44,49,54,61,62,68,71,75,80], the Fitbit app (9/68, 13.2%) [16,31,37,40,67,75,77,80,84], and Noom (9/68, 13.2%) [16,28,31,52,67-70,73]. The process used to extract individual apps is illustrated in Figure 3, while Table 3 presents the 46 apps along with their available establishment year, country, and a brief description.

**Table 3:** Description of the 46 single apps included in the retrieved studies.

App name: study	Established year	Country	Short description
Akser Waznk; Alturki and Gay [22]	—^a^	Saudi Arabia	The app was developed in Saudi Arabia and assists users with obesity in adopting a healthy lifestyle through motivation and dietary tracking.
Argus; Ferrara et al [23]	2012	United States	The app tracks activity, dietary intake, and sleep to support weight management.
Bitesnap; Gioia et al [24]	2017	United States	The app helps users track food intake and manage their diet through a visual approach.
Cronometer; Gioia et al [24]	2005	Canada	The app provides detailed nutrition tracking and calorie-counting to support weight management.
DialBetics Lite; Kondo et al [25]	—	Japan	The app records diet, physical exercise, and diabetes self-management data.
DietLens; Tahir and Loo [26]	2018	Singapore	The app uses AI^b^ to identify foods and estimate nutritional composition from images.
Doctor Diary; Kim et al [27]	—	India	The app tracks calories and diabetes-related data and enables monitoring by health care professionals.
Easy Diet Diary; Chen and Allman-Farinelli [28]	2012	Australia	The Australian app provides calorie-counting and dietary tracking with physical activity monitoring.
EatsUp; Agustina et al [29]	—	Indonesian	The Indonesian app records dietary data and recommends menus based on caloric targets.
ENGAGED; Pellegrini et al [30]	2012	United States	The app was developed as part of the ENGAGED trial, allowing for dietary self-monitoring and calorie counting, as well as physical activity tracking.
FatSecret; Ferrera et al [23]	2007	Australia	A free food diary app that allows users to log the amounts and types of foods and beverages consumed, providing nutritional information, including calorie estimates.
FDDB extender app [86]	—	Germany	The app assists users in tracking calories for weight reduction or maintenance.
Fitbit; Burke et al [31]	2007	United States	The app tracks calories, diet, and other health-related metrics.
Food Tracker; Tahir and Loo [26]	2019	Canada	The app logs food intake and monitors nutritional consumption for health management.
FoodCam; Raju and Sazonov [32]	2015	United Kingdom	The app uses image recognition to identify foods and provide nutritional information.
FoodLog; Lemacks et al and Aizawa et al [33,34]	2013	Japan	The app tracks meals and monitors nutritional intake.
Fooducate; Chaudhry [35]	2010	Israel	The app helps users make healthier food choices by providing nutritional insights.
GoCarb; Anthimopoulos et al [36]	2016	Switzerland	The app estimates the carbohydrate content of meals from food images for diabetes management.
Good Measures; Olfert et al [37]	—	United States	The app is designed to support healthy food choices and behavior change related to eating and exercise via food entry and nutritional balance evaluation.
iDAT [38]	—	Singapore	The app is designed for the Singapore population. Functions as a calorie counter, helping users to balance calories consumed with calories burned daily.
Im2Calories; Myers et al [39]	2015	Japan	The app uses deep learning to estimate calorie content from food photographs.
January AI; Zahedani et al [40]	2023	United States	The app integrates CGM^c^ and HR^d^ data with user-entered diet and activity data.
Keenoa; Moyen et al [41]	2020	Canada	The app uses image-assisted food diaries with AI-based food recognition.
KIT-Nutrition app; Schusterbauer et al [42]	—	United States	The app combines dietary self-monitoring with diabetes management features.
LIBIT; Oh et al [43]	—	Korea	The app records diet and exercise, connects to home devices, and provides feedback.
Lifesum; Ferrara et al [23]	2013	Switzerland	The app combines calorie counting with healthy lifestyle recommendations.
Lose It!; Burke et al [44]	2008	United States	The app allows users to record daily food intake and physical activity for weight loss.
Menu-Match; Beijbom et al [45]	2015	United States	The app helps users with dietary restrictions find suitable restaurant meals.
Mobile food record (mFR); Ahmad et al [46]	2021	United Kingdom	The app captures before-and-after meal photos to assess dietary intake and patterns.
MyDietCam, Tahir and Loo [26] and Chui et al [47]	2020	Malaysia	The app analyzes meal photos to provide nutritional information and personalized diet advice.
MyFitnessPal; Tosi et al [48]	2005	United States	The app tracks foods and beverages to provide nutritional information and calorie analysis.
MyMacros+; Gioia et al [24]	2014	United States	The app tracks macronutrients for weight management.
MyMealMate; Carter et al [49]	2012	Australia	The app facilitates weight loss with an electronic food diary for dietary self-monitoring.
MyNetDiary; Fu et al [50]	2007	United States	The app provides personalized weight loss advice and tracks food intake.
MyPlate by Livestrong; Ferrara et al [23]	2004	United States	The app tracks calories and nutrients and supports diabetic users.
nBuddy Diabetes (Nutrition Buddy Diabetes); Lim et al [51]	2017	Singapore	The app supports diabetes self-management through meal logging and activity tracking.
Noom; Jin et al [52]	2008	United States	The app combines calorie tracking with behavioral coaching for weight management.
Nutritionix; Kay et al [53]	—	United States	The app enables self-monitoring of dietary intake for daily nutrition tracking.
PlateMate; Zhou et al [54]	2011	United States	The app estimates the nutritional content of foods from photos using crowdsourcing.
SAlBi (Salud, Alimentación, Bienestar, and educación); Gonzalez-Ramirez et al [55]	2022	Spain	The app provides self-monitoring and tailored dietary advice based on the Mediterranean diet.
Snap-n-Eat; Zhang et al [56]	2015	United States	The app automatically recognizes food and estimates calorie and nutrient content.
SparkPeople; Bardus et al [57]	2001	United States	The app tracks diet, exercise, and weight changes over time.
T1DEXI; Riddell et al [58]	2020	United States	The app tracks food intake and exercise for diabetes management.
Unnamed prototype app	—	United States	The prototype app provides real-time feedback on diet quality and heart disease risk.
WeightWatchers (WW); O’Neil [59]	1963	United States	The app uses a point system to promote healthy eating and weight loss.
Yazio; Puigdomènech et al [60]	2013	Germany	The app assists with calorie counting and diet planning for weight loss.

^a^Not available.

^b^AI: artificial intelligence.

^c^CGM: continuous glucose monitoring.

^d^HR: heart rate.

### Synthesis of Results

The data extracted from the 68 papers were categorized into four dimensions: (1) features and functionalities of calorie-counting apps, (2) acceptability, (3) feasibility, and (4) adherence. These dimensions are presented below in more detail.

#### Features and Functionalities

##### Logging-In

When beginning to use an app, it may ask the user to provide personal information, such as an email, to allow the user to sign up and create an account to use the app. This allows for information on the mobile device to be saved as the user uses the app, linking the user’s information to their account. The review of papers underlined two methods for the storage of information on an app: the creation of a new account on the app’s software itself or signing in by linking to an already existing social media account. Of the 46 total apps discussed, 16 (35%) were said to have a login feature. One additional paper discussing this feature was asked not to specify the app portrayed. Doctor Diary [27], Akser Waznk [22], and Lose It! [61] were apps that were mentioned to use email addresses as a means of signing up to the platform. Akser Waznk was also described as offering the option of linking to a Facebook, Instagram, or X (formerly known as Twitter) account for users to sign up to the app [22].

Additionally, papers also described the process of entering personal and health information in order to accurately customize goals and recommendations. Akser Waznk [22], Lose It! [62], and Noom [52] were reported to ask users to enter their gender, age, height, and weight at the time of sign-up. Additionally, Akser Waznk had an optional step of adding health information, such as the user’s medications and chronic diseases [22]. Doctor Diary [27], MyFitnessPal [63], and FDDB extender [64] had mentions of a login feature requiring user information, but no further description was provided. The 18 other apps had no mention of the logging-in process, according to this study, although it is possible that this feature is present in these apps and has yet to be described.

##### Calorie-Counting Features

When determining the acceptability of an app, the way that the process of calorie counting is done is crucial [65]. On that same note, an app that allowed for fast analysis of calorie content (meaning that the user needed to spend as little time as possible to log their calorie intake, as well as the ease of use of the said feature) was generally viewed as favorable [66]. Among the 68 papers, 17 (25%) included more than one app or did not mention the availability of calorie-counting functionality. The remaining 51 papers described 3 main methods of calorie counting: manual entry, picture-based entry, and barcode scanning.

Manual entry was generally described as the ability of the user to enter food products to get an estimation of total calories (ie, diary data entry). A total of 44 out of the 46 (95.6%) apps used calorie manual entry. Although the general concept of manual entry is well described, there is one significant difference when it comes to the functionality of calorie estimation: the integration of a food database. These databases, which could include up to 6 million foods within the app [67], provided a base of caloric information to be retrieved when users logged their consumption and removed the need for users to enter the calorie counting themselves. The databases could also be tailored to match certain population demographics (17/46, 37% apps had country-specific food databases). Once a food item was found, the user quantified the amount consumed. Of the 44 manual entry method apps, 26 (56.5%) had the use of a food database mentioned within their respective paper. Finally, it is worth noting that LIBIT had a voice recognition feature for logging meals [43].

**Figure 4:**
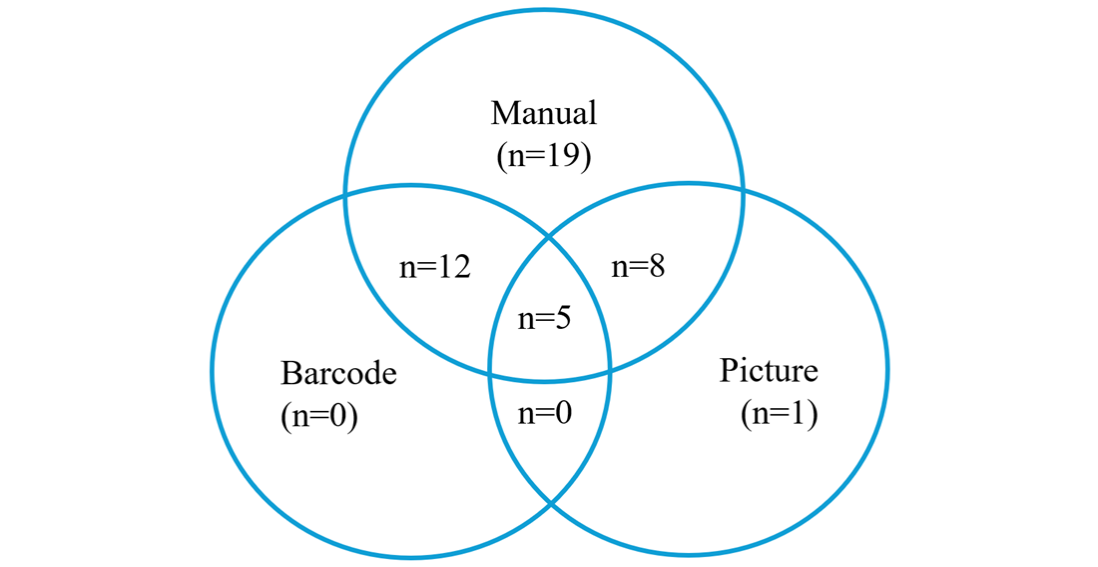
Venn diagram shows overlap of calorie-counting methods used by apps.

Picture-based entry was described as the process of taking a picture of a food before consumption to get a calorie count estimation by the app. This was usually done through an integrated software that recognizes food items [62]. A total of 14 (30.4%) of the 46 apps mentioned this method of calorie counting (Figure 4).

The last method of calorie counting was through barcode scanning. This method relied on scanning barcodes of store-bought food items, which allowed the app to identify food items, and the user then logged the quantity consumed to get a calorie intake estimate [64]. A total of 17 (36.9%) of the 46 apps were mentioned to have this feature.

It is important to mention that certain apps include multiple methods for calorie counting. A total of 17 apps had both manual entry and barcode entry, while 5 apps had a combination of all 3 methods. These relationships are portrayed in Figure 4.

##### Goal Setting

Another feature that was often described within the calorie-counting apps designed for individuals living with chronic health conditions was the ability for users to set goals within the app. Indeed, among the 46 individual apps discussed in papers, 24 (52.2%) had a form of goal setting. While the specific type of goal could vary in composition, the most commonly described is one that calculates a calorie budget or allotment via the input of a weight loss goal determined by the user, as well as the input of certain characteristics such as current weight, height, and physical activity level (usually done when going through the account setup). One method by which this caloric budget calculation was made was through the Harris-Benedict equation [29]. Goal setting in the form of a weight loss target and a caloric budget was clearly described by Wharton et al [68] and was found to be the most common type of goal setting since it is used by 17 of the 24 apps (70.8%) that have a goal setting functionality.

Of the remaining 7 apps, 6 had goal settings mentioned without specifying the way it was integrated, and 1 was mentioned to have goal setting in the form of nutritional balance, which consisted of encouraging users to preset a goal of servings per food category, such as fruits and vegetables [69].

##### App Interaction With User

Some mobile apps could interact with the user to encourage use of the app and its functionalities (ie, tracking). These interactions could be done while the user was actively using the app (synchronous) or when the app was not in current use (asynchronous), such as providing reminders to the user. Of the 46 total apps, 30 (65.2%) mentioned in the papers interacting with the user by using reminders or by providing intake recommendations and feedback. Notably, MyFitnessPal [67], Akser Waznk [22], Noom [70], EatsUp [29], nBuddy Diabetes [51], Lose It! [44], January AI [40], Keenoa [41], WeightWatchers (WW) [59], FatSecret, Cronometer [24], Yazio [60], MyPlate by Livestrong, Lifesum, SparkPeople, Argus, and MyMealMate [49] used reminder messages to log intake or to increase adherence to recommendations. Eisenhauer et al [71] found that text messages, such as Lose It!, would improve self-monitoring in users at 3 and 6 months. While the presence of reminder functionalities was extracted, the frequency or timing of these reminders was not consistently reported across studies and, therefore, was not included in the data synthesis.

Interactions also consisted of providing feedback and health information to the users. Doctor Diary [27], MyFitnessPal [67], DialBetics Lite [25], SAlBi educa [55,72], Nutritionix [53], January AI [40], LIBIT [43], EatsUp [29], MyMealMate [49], and Good Measures [37] provided feedback on nutrition. This consisted of proposition suggestions on energy intake and food recommendations. Gonzalez-Ramirez et al [55] stated that users found the tailored feedback messages to be one of the most useful features used in the SAlBi educa app and that 86.1% of users believed this feedback would have a positive effect on user diet, while Kay et al [53] stated that only 55% of users believed this feedback was useful in the Nutritionix app. Nutrition feedback was also conducted in different manners other than live in-app messages, such as providing health-related papers in Noom [73] or educational videos on nBuddy Diabetes [74]. Some apps also displayed the logging of calories in visual feedback for the users, which allowed a more comprehensive way of showcasing their data. Easy Diet Diary [28], MyFitnessPal [67], Lose It! [75], Fitbit [31], ENGAGED [30], SAlBi educa [55], and MyMealMate [49] were apps that mentioned presenting the user’s intake data in graphs or charts, according to the select papers.

##### Other Functionalities

Most of the apps studied in the selected papers had multiple functions other than calorie counting, for example, weight or blood glucose monitoring. Other key features described in the literature were related to the ways users can log health information. These key features included remote patient monitoring by health care providers, graphical or visual data, and sharing progress with other users. Some apps included information on recipes and color schemes, as well as the possibility to upgrade to a premium version (with more features and functionalities). Out of the 46 total apps, 34 (74%) were mentioned to have some of these features. Doctor Diary [27], EatsUp [29], LIBIT [43], Good Measures [37], MyFitnessPal [76], Lose It! [71], SAlBi educa [55], January AI [40], MyMacros+, Lifesum, SparkPeople, Argus, and WeightWatchers (WW) [13] had features other than calorie counting for users to track, such as weight tracking or glucose monitoring, according to the literature retrieved.

Sharing the user’s progress with health care providers could be done in real-time tracking or later, providing insight into the user’s tracking habits. A total of 15 (32.6%) out of the 46 total apps included this feature. Doctor Diary [27], Noom [70], EatsUp [29], Easy Diet Diary [28], nBuddy Diabetes [51], FDDB extender [64], LIBIT [43], Nutrinaut [42], Good Measures [37], Cronometer, MyNetDiary, WeightWatchers, Lifesum, MyPlate by Livestrong, and SparkPeople were described as apps that allowed health care providers to access the user’s tracking data. Where specified, health care providers involved were physicians and registered dieticians.

An additional key feature discussed for 15 (32.6%) of the 46 apps was the ability for users to have real-time chats with other users or to share their progress on the app for others to see. Lose It! offered discussion boards that would have benefited the users more if they were done in a synchronous manner, which would have increased the feeling of support, according to participants [71]. Akser Wankz and WeightWatchers also provided features for users to chat with others about their progress [22]. Other apps like iDAT, Yazio, Lifesum, SparkPeople, Argus, WeightWatchers, and Fooducate allowed users to share their progress directly to a social media platform, Facebook [78]. MyFitnessPal’s networking feature was assessed, and 80% of participants reported having “no friends” on the app, which highlighted the minimal use of this feature [79].

Color schemes were also discussed in select papers, providing an insight into the importance of aesthetics in applications. Out of the 46 total apps, 13 (28.3%) had discussions on the aesthetics used. Both EatsUp [29] and Akser Waznk [22] were discussed in terms of their use of color schemes. The use of colors allowed for an increase in appeal and encouraged the use of apps. According to Alshathri et al [80], MyFitnessPal, Fitbit, and Lose It! were also some of the highest-ranked apps in engagement and aesthetics.

Some apps also had the option of premium access, allowing the user to pay for more advanced features not included in the free version of the app, such as Noom [73], MyFitnessPal [81], and Lose It! [62]. In general, 13 (28.3%) of the 24 apps mentioned have a premium option, which offers exclusive functions with enhanced support and patient monitoring. Although premium features were available, most participants expressed a preference for free apps, with cost emerging as one of the primary factors influencing their app selection [64].

Overall, free, of charge, and self-explanatory apps were most preferred by users. Sharing their progress with others or through a platform were features deemed to be less important by users [66].

##### Validity, Accuracy, and Reliability

Calorie-counting apps that were available to the public used different methods to log calories and could provide recommendations to users for healthier options. For reliability, these apps could rely on evidence-based theories, use known food databases, or be developed by a team of health care professionals. Of the total 46 apps, 22 (47.8%) were mentioned in papers to use at least one of these methods. EatsUp [29], Nutritionix [53], Kit-Nutrition [42], and SAlBi educa [55] were apps mentioned in the retrieved literature to have been developed or maintained by health care professionals, such as dieticians or physicians.

As discussed previously, food databases were sometimes used by the apps to correctly estimate calorie counting for the user. This thereby provided reliable, accurate information to the users. Easy Diet Diary was described to use the Australian Food and Nutrient Database 2011-2012 [28] while FDDB extender used the FoodData Central of the US Department of Agriculture and the Souci-Fachmann-Kraut databases [64]. Additionally, SAlBi educa used the BEDCA database for reference during food logging [55]. January AI was also said to use known databases, but this was not further detailed in the chosen literature [40]. On the other hand, Nutritionix itself was highlighted as the largest verified database for nutrition information by [53].

In terms of evidence-based theories, these apps were mainly developed to manage chronic diseases, such as obesity or diabetes. They could thereby rely on chronic disease management or behavioral theories to develop an effective calorie-counting app, like the social cognitive theory. Akser Waznk [22], EatsUp [29], MyMealMate [82], SAlBi educa [72], and Good Measures [37] were apps that were described to have relied on evidence-based theories on nutrition or chronic disease management. Lee et al [73] rated Noom the highest score of information accuracy, but the paper did not provide additional information on the theories used to develop the app. Keenoa was evaluated to have moderate to strong relative validity compared to an Automated Self-Administered Dietary Assessment Tool, but more validity establishment was needed [41]. Table 4 summarizes the main identified features and functionalities, along with the number of papers where they were presented and a short description.

**Table 4:** Description of the identified features and functionalities.

Feature	Apps with the feature mentioned (N=46), n (%)	Description
Logging in	16 (35)	Logging in to the app allows users to save information. The sign-up process may require personal or health information.
Calorie counting	45 (98)	There are 3 general ways by which apps count calories: manual entry, barcode scanning, and picture-based entry. Almost all apps have some form of manual entry, which is facilitated by the integration of a food database in most cases. A total of 5 apps offer a combination of methods.
Goal setting	24 (52)	Goal setting is generally available in the form of a weight loss goal, which allows the app to calculate a caloric budget. One app offers the setting of nutritional balance goals.
App interaction with the user	30 (65)	Interactions consisted of reminders to increase app use or the provision of feedback and recommendations on nutrition.
Other	Other functions of the app: 34 (74) Monitoring by health care providers 15 (33) Premium subscription: 13 (28) Data sharing: 13 (28) Visual aesthetics: 13 (28)	Most apps have a combination of calorie counting and other functionalities, including information exchange with health care professionals or other patients, data visualization, and an upgrade to a premium version.
Validity, accuracy, and reliability	22 (48)	To be reliable, some apps were developed according to evidence-based theories, such as behavioral theories. Other ways of ensuring reliability include health care professionals in the development of apps or using known food databases for accurate nutritional information.

#### Acceptability

From the scoping review database, we extracted the factors that favor or limit the acceptability of calorie-counting apps from the perspective of users and of health care professionals. When identifying factors contributing to acceptability, we used the following definition as provided by Garizábalo-Dávila et al [83]: “Acceptability is the perception of patients and health professionals versus determining whether the intervention is appropriate to address the problem, in a reasonable, adequate, and convenient manner for its application in daily life.” We summarized below the key findings according to the various structural, cultural, socioeconomic, behavioral, quality standards, and ethical factors identified.

#### Structural Factors

Various characteristics of the apps’ format, features, functionalities, and content influenced their acceptability from the users’ perspective (Textbox 2). The scoping review showed that features or functionalities personalized according to users’ preferences and characteristics increased the acceptability of calorie-counting apps. For instance, users were very satisfied with the possibility to choose color schema and app themes through a variety of options [22]. Users also liked the feature of allowing them to highlight the nutrients of most importance to them in the app when tracking their dietary intake [28]. The personalization of reminders or feedback generated by the app improved acceptability. Receiving app recommendations based on previous successes or failures to meet dietary goals [65] or getting a personalized progress report each week [84] are examples of functionalities that increased users’ satisfaction.

**Facilitators of acceptability of calorie-counting apps.**

**Format (structural)**

- Esthetically pleasing design
- Streamlined interface
- Ease of carrying a smartphone
- Paperless food record

**Features (structural)**

- Personalized features
- Self-monitoring features
- User-friendly screen features
- Food image
- Technical support from the app provider (data derived from both users and health care professionals)

**Functionalities (structural)**

- Augmented reality
- Automatic calorie or nutrient estimation
- Automatic food recording
- Integration with other apps or platforms
- Sharing information with a health care professional, family, or on social networks
- Ability to monitor goals
- Useful and personalized reminders or feedback

**Content (structural)**

- Availability of varied food items
- Appropriate and clear terminology
- Appropriate translation
- No need for training
- Availability of support or training

**Cultural**

- Health awareness (data reported exclusively by health care professionals)

**Socioeconomic**

- e-literacy
- App free of charge
- Gender
- Rurality

**Behavioral**

- Sense of accountability
- Motivation
- Performance expectancy
- Habit
- Anxiety risk

**Quality standards**

- Validation
- Certification

In Textbox 2, all items represent data reported by participants, unless indicated.

Automated functionalities and the possibility to share app data through well-integrated platforms increased app usability or usefulness and increased users’ acceptability (Textbox 2). For instance, users enjoyed an app including automatic calorie or nutrient estimation [66] and automatic food recording through barcode readers, meal image analysis, or artificial intelligence-based food detection [65,66,69]. On the contrary, manual entry of food consumption represented a major deterrent to using a calorie-counting app [85]. Users were satisfied with the possibility to share data recorded on the app with health care professionals, family members, or on social media. Because saving and sharing dietary record history was not available on all calorie-counting apps, the absence of this functionality was also a factor hampering acceptability. Users also noted that the functionalities of some apps were limited by bugs, crashes, and insufficient updates, and these factors all reduced acceptability.

The self-monitoring features were considered by users to be useful components of an app. Being able to daily track food intake [84], to easily log dietary intake with copy-paste functionalities [28], or to easily input beverage count [29] was appreciated. Users were also motivated by the ability to monitor goals or to fill out an action plan weekly [84]. Being able to visualize a weight chart showing the initial, current, and goal weight on a progress screen on some apps represented a great source of satisfaction for users [22]. These self-monitoring features, being available as paperless food records on an easy-to-carry device such as a smartphone, were also noted by users as a strength in terms of usability [29].

In terms of interface and content, user-friendliness was highlighted as an important characteristic of a calorie-counting app. Users’ satisfaction increased with the presence of self-explanatory features that did not require training [66] or when users could easily find all subscreens on the main screen [22]. With respect to recording food consumption, the availability of a variety of food items represented a clear strength for an app [37]. This was especially true when app designers chose to use food images over text as an option to select dietary items [29]. On the contrary, the acceptability of an app decreased when users were unable to find specific food items on the app, either because they were unavailable or difficult to find. More specifically, many papers highlighted that an app’s usefulness decreased when lacking multiethnic, local, and home-cooked foods as well as take-out meals and restaurant options [28,37,65,79,81]. In addition, confusion on the app with respect to portion size was another barrier to acceptability. Finally, users noted the importance of clear terminology [72] and appropriate translation [81] in calorie-counting apps. Nutritional messages that were easily understood by users with a lower level of education were also identified as a positive factor of acceptability [72]. Finally, users’ satisfaction was higher when the app design was aesthetically pleasing, for instance, when it used attractive color schemes, an appropriate font size, and a streamlined interface [79].

#### Other Factors

Among cultural factors, health awareness was recognized by both users [37,74,78,81] and health care professionals [22] as a factor that increased the acceptability of an app. In parallel, some users identified that they were not comfortable sharing their electronic dietary record with a health care professional, and this limited the usefulness of the app [28].

The scoping review identified many socioeconomic factors contributing to the acceptability of calorie-counting apps. The most frequently cited factor was e-literacy. In fact, some apps were considered user-friendly, but only for users familiar with smartphone apps [28]. One study identified that this could be especially true for older patients [75]. The influence of the level of e-literacy on the acceptability of calorie-counting apps was not restricted to users, as one study reported that health care professionals with low levels of e-literacy could also lack the skills to efficiently use or counsel patients using calorie-counting apps [28]. Financial aspects were also addressed as factors influencing acceptability. Free apps had a higher level of acceptability [66], but not being able to afford a smartphone was a major barrier to app usability [37]. In addition, the usefulness of certain recommendations generated by apps varied with the type of work schedules. For instance, shift workers reported that, depending on their shift schedule during a given week, they were not always able to meet fixed weekly goals included in recommendations automatically generated by the app [65]. Sex affected adherence to app use, as females were more consistent users compared to males [78]. Finally, participants living in rural areas compared to those in urban areas shared more favorable views of calorie-counting apps [37].

Behavioral characteristics of users could affect the acceptability of calorie-counting apps. For users who mentioned the importance of being kept accountable, the usefulness of using a calorie-counting app increased when combined with the oversight from a dietician [28]. In 2024, Chew et al [74] showed that the intention to use AI-assisted weight management apps is influenced by factors such as age, anxiety risk, and the desire to maintain a healthy diet. These factors together explain a significant portion of why people might choose to use these apps. However, self-regulation, depression risk, BMI, and waist circumference do not seem to affect the decision to use AI-assisted weight management apps.

In terms of quality standards, validation and certification of an app represented criteria for selecting a nutrition and diet app by users [66]. Although automated functionalities in an app increased its acceptability level as addressed above, the lack of accuracy of automated functionalities led to user frustration [69]. Finally, for dieticians, a key barrier to using calorie-counting apps was the lack of confidence associated with the reliability of sharing patients’ personal information [28].

In Textbox 3, all items represent data reported by participants, unless indicated.

**Barriers to the acceptability of calorie-counting apps.**

**Functionalities (structural)**

- Saving and sharing history and favorite not supported
- Bugs, crashes, or server connectivity
- Insufficient app updates
- Manual entry of food consumption
- Impossibility to edit and delete entered values
- Reliability of data sharing unknown (data reported exclusively by health care professionals)

**Content (structural)**

- Demotivating reminders
- Unavailability or difficulty in finding food items
- Confusion with portion sizes

**Cultural**

- Discomfort with sharing app data with a health care professional

**Socioeconomic**

- Internet unavailability
- Low level of e-literacy (data derived from both users and health care professionals)
- Older age
- Smartphone availability
- Work schedule

**Quality standards**

- Automated techniques not accurate
- App not validated (data derived from both users and health care professionals)
- App not certified (data derived from both users and health care professionals)
- Confidentiality of data sharing is unknown (data reported exclusively by health care professionals)

#### Feasibility

Barriers that affected the feasibility of the apps in a clinical context were classified into 5 categories: structural, cultural, socioeconomic, behavioral, and accuracy (Textbox 4).

**Barriers to feasibility from the patient (or health care professional) perspective.**

**Structural**

- App crashes
- Server connectivity
- Technical problems
- Calorie logging difficulties
- Limited smartphone platform
- No barcode scanning option
- Time consuming

**Cultural**

- Limited food database

**Socioeconomic**

- Web version of the mobile app
- No compatible smartphone

**Behavioral**

- Food recommendation
- Portion estimation by the user

**Accuracy**

- App underestimating calories
- App overestimating calories

#### Structural

It was not unusual for the users to experience app crashes, technical problems, and difficulty with server or internet connectivity [42,79,85].

Some apps did not contain the barcode scanning feature or needed the internet for the entry of food items, which then caused loss of motivation [22]. Logging food and beverage intake in an app could be effortful and time-consuming, taking an average of 15 to 20 minutes per day, and behavioral fatigue sometimes resulted [42,87]. On a similar note, one study found that dieticians thought the Easy Diet Diary was time-consuming to monitor and made it difficult to review patient records in a timely manner. The issues created a loss in motivation to keep using the app [28].

#### Socioeconomic

Some participants did not have a smartphone, or the smartphone was not compatible with the app [22,42]. Lose It! had a smartphone and web-based version, although the web version could be limiting since it did not contain the same features. For example, the photo feature of the app was not available through the web version [62].

#### Behavioral and Cultural

Users did not always accurately estimate food portions and nutritional contents [69]. Food databases were available to help users, but the databases could be quite large for the calorie-counting apps, and could be missing certain food products or recipes, especially when it came to more regional food items and homemade meals [41,42,64,72]. Capacity and accuracy of the search engine and the variety of meals and food terminology are key aspects to consider when food databases are created.

#### Accuracy

In a prospective controlled trial that assessed the quality and effectiveness of popular calorie-counting apps in weight management and behavior change, calorie and activity recommendations were compared with standards, and over 65% of apps over- or underestimated calorie intake [85]. Recent evidence further emphasizes that incorporating structured behavior change strategies within mobile app interventions can significantly enhance their effectiveness in improving weight-related outcomes [88].

#### Adherence

All calorie-counting apps saw a decline in adherence over time, although both the definition and level of adherence differed from one study to another. Definitions ranged from logging total daily dietary intake to simply using the app at least once a day, with some studies requiring a minimum number of calories to be recorded. Despite varying definitions of adherence, studies consistently showed that adherence declined over time, with several reporting a sharp drop, in some cases to nearly zero, after the intervention period. Adherence levels were typically measured by collecting the app data, which tracked how frequently and how much information participants entered. However, recent findings suggest that strong early app engagement may predict better long-term adherence and greater weight loss outcomes [89].

Notably, some apps had good adherence in the first few months before decreasing. One study reported that participants self-monitored with MyFitnessPal an average of 5.4 days/week during the first 4 weeks, declining to 1.4 days/week during weeks 5-12, and to 0 days/week after the 12-week intervention [67]. Another study in Doctor Diary found that 14/32 participants (43.8%) failed to record three daily meals throughout the 8-week period, meaning only 18/32 participants (56.2%) were considered adherent based on the study’s criteria [21].

Comparingly, a study on “Lose it!” found that in the first 6 weeks, participants used the app daily but decreased to 4 days per week at the end of the 12-week study [75]. Multiple studies showed that Lose It! had high adherence compared to a food journal group or an electronic diary group [31,61,68]. In one study that evaluated the adherence of MyMealMate, calorie counting declined over time so that by 6 months, 7 of the participants (16%) in the smartphone group had managed to record their dietary intake every day, but no participants in the food journal and web group had achieved this [49]. The following section outlines 4 factors found to enhance adherence, also summarized in Textbox 5.

**Factors that facilitate adherence are divided into 4 categories: structural, user-related, financial, and environmental.**

**Structural**

- Photo feature
- Paired with a structured program
- Paired with a Facebook group
- Simplified monitoring

**User-related**

- Health awareness
- High motivation by the participant
- Higher education

**Financial**

- Monetary incentive
- Access to the premium version

**Environmental**

- Internet access

#### Socioeconomic Factors

In a study comparing premium versions of apps to free versions, they found that premium versions had a higher adherence rate, defined as logging of 800 daily calories or more [71]. Although another study with MyFitnessPal Premium also confirmed that the adherence to logging all meals 4 or more days a week still decreased over time, from 39% to 8% to 0% at week 1, 12, and 24, respectively [81].

One study examined the effectiveness of a monetary incentive with a deposit system with the goal of motivating participants using Noom. With this in place, more than half of the participants completed the 16-week program by logging three or more times a day [73].

#### User-Related Factors

Adherence to daily app use was found to be associated with good comprehension of the impact associated with their health condition and the desire for weight loss [85]. Similarly, low motivation for habit change was associated with low daily adherence [78,85].

In terms of specific user demographics, a study on Good Measures noted that adherence to daily meal-logging in the app did not vary between peri-urban and rural sites [37]. In another study, participants with a higher level of education had a higher general adherence to the app [53].

#### Structural Factors

Some features were found to have increased participants’ adherence to the app. For example, a retrospective cohort study evaluating Lose It! showed that the group who used the photo feature by taking a snapshot of their food items and selecting the right items from a list of suggestions to display calories had 6.1 more total logged days than those who did not use this feature [62]. This remained significant after adjusting for user demographics such as age, gender, and BMI.

One way to increase adherence was to pair the app with a structured program or a Facebook group [31,81], but another documented way is to simplify calorie counting. For example, monitoring only the high-calorie foods consumed that have limited nutritional value. In a study comparing detailed counting of calories on Fitbit to simplified calorie counting, the group with the simplified option had better adherence throughout the study, with a median day of self-monitoring of 97% compared to 49% for the detailed group [84].

#### Environmental Factors

The need for an internet connection to manually enter food intake in the app was negatively associated with daily food logging adherence [85].

## Discussion

### Recommendations

Based on the results of our scoping review, we provided a list of recommendations to improve acceptability and feasibility associated with the use of calorie-counting apps to monitor and manage weight in adult populations living with obesity and weight-related chronic disease. We present below our recommendations for developers, clinicians, and researchers (Table 5), based on potential improvements associated with behavioral, structural, socioeconomic, and research and development components of the use of calorie-counting apps. These recommendations are a result of collecting information on the functionalities, features, and user engagement metrics that factored into the acceptability and feasibility of the apps.

**Table 5:** Recommendations to improve the acceptability and feasibility of implementing the use of calorie-counting apps in clinical practice in populations living with weight-related chronic disease.

Category and subcategory		Recommendations
**Behavioral**		
	Motivation	Ensure a thoughtful frequency of reminders, feedback, and progress reports to help users stay on track without inducing fatigue or discouragement Allow users to opt out of notifications to avoid discouragement in certain users Include game features in apps, deliver app-based interventions in a context of group competition, and/or provide financial incentives associated with app use to increase motivation
	Guidance	Provide more guidance on how to make healthy food choices and how to deal with diet slips to help users progress toward their goals
**Structural**		
	App function	Ensure regular app updates to stop the frequent app crashes and decrease frustration from app users Allow offline functioning to improve consistency of calorie logging Offer users simplified calorie logging options, such as only keeping track of high-calorie and low-nutrition food, to reduce the workload associated with calorie tracking
	Content	Include a variety of multiethnic, local, and home-cooked foods as well as take-out meals and restaurant options for users to choose from when logging dietary intake Include features adapted for users with low vision, such as simplified terminology and screen organization, as well as clearly defined tabs Include a tutorial with the app to make sure users understand all functionalities
**Socioeconomic**		
	Costs	Offer a free or very affordable app when implementing an app used in clinical interventions to avoid reduced accessibility for populations with low income
**Research and development**		
	Engagement	Involve future users and health care professionals in all stages of app development and clinical interventions, as well as in associated research projects, to foster co-construction of technological and clinical approaches

#### Behavioral Recommendations

##### Motivation

To develop a calorie-counting app that is well-suited for clinical implementation, it is important to consider some factors that keep users engaged. Motivation is one of the major factors fostering long-term app use and thus promoting adherence. Our scoping review showed that reminders, feedback, encouragement, and progress reports were well received by users (see the Acceptability section). For the best results, they should be thoughtfully spaced out so that they help users to stay on track without being too frequent to avoid fatigue or discouragement. This is especially true for the progress reports, where too frequent reports can lead to stagnant results and loss of motivation. In addition, allowing users to opt out of notifications to avoid feeling discouraged represents another associated recommendation. To increase motivation to use calorie-counting apps, other behavioral recommendations have been suggested, such as including game features in apps, delivering app-based interventions in a context of group competition, and providing financial incentives associated with app use [79].

##### Guidance

Our scoping review suggested that greater user understanding of calorie intake and its health implications may be linked to better engagement with calorie-counting apps [85]. Accordingly, providing structured guidance on healthy food choices and strategies to address dietary lapses could support user adherence and long-term behavior change.

#### Structural Recommendations

##### App Function

We formulated various recommendations related to the app function. First, ensuring regular app updates to stop the frequent app crashes would decrease frustration from app users. In addition, having an offline option would enable users to be more consistent by tracking calories when they do not have internet access. To reduce the workload and time spent tracking calories, we recommend offering users simplified calorie-counting options, such as only keeping track of high-calorie and low-nutrition food, for instance. Another recommendation to improve function while reducing workload for users would be to rely on artificial intelligence to perform automatic estimation of the calorie content of food items shown in pictures. This could significantly simplify the task of calorie logging for users and hence increase motivation and adherence.

##### Content

The content of calorie-counting apps could also be improved. To reduce workload for users and increase app usefulness, we recommend including a variety of multiethnic, local, and home-cooked foods, as well as take-out meals and restaurant options for users to choose from when logging into dietary intake. Moreover, app content should reflect diverse cultural and racial backgrounds to ensure inclusivity and relevance across populations with different dietary habits and nutritional needs. In addition, to tailor the app for users with low vision, we recommend the use of simplified terminology and screen organization with clearly defined tabs that can be easily seen. Finally, including in the app content a tutorial for users to understand all the functions the apps have to offer also represents a key recommended improvement for calorie-counting apps.

#### Socioeconomic Recommendations

Our scoping review identified that the cost of a calorie-counting app could represent a barrier limiting its acceptability and, consequently, the adherence to the app. We recommend offering a free or very affordable app to improve accessibility for users with lower income, thus making it easily recommendable by health care professionals to all patients.

#### Research and Development Recommendation

Successful implementation of calorie-counting apps in clinical interventions targeting patients living with weight-related chronic diseases relies on reaching high enough acceptability, feasibility, and adherence levels for both users and health care professionals. For that purpose, we provide a general recommendation to foster engagement of these stakeholders in all stages required to develop an app, but also to implement app use in clinical practice. Co-construction of the technological and clinical approaches together with users and health care professionals would be instrumental to strengthening acceptability, feasibility, and adherence.

### Limitations

Given our search strategy, which required papers to include populations living with chronic conditions, studies that only described apps without targeting these populations were not necessarily included. This may have limited the depth of our features and functionalities of calorie-counting apps analysis. Even when combining app descriptions from the included studies, it is possible that some features were missed if the primary objective of certain papers was not to describe the app in detail. We acknowledge that this may have led to the omission of specific app features. Nevertheless, we believe that our study still achieved its objective of providing an overview of the most common design features of calorie-counting apps for adults living with chronic conditions. Another limitation of this review is the lack of consistent reporting on reminder frequency within the primary studies, which prevented analysis of how reminder timing or intensity might influence user engagement.

### Implications for Future Research

Building an app with these recommendations in mind will be a crucial step toward developing an application that meets the needs of adults living with chronic conditions in clinical settings. Another essential step in the development of these applications is the need for future research. As is the case with any intervention destined for a clinical setting, the validity of these apps, as well as an evaluation of their effectiveness, should be documented through future research. Many of these applications have a goal of promoting weight loss and healthy eating behaviors, and the effectiveness with which they can achieve those goals should be studied. As was done throughout the development of many studied apps in the papers selected by this study, future research should also evaluate the acceptability and the feasibility of the app throughout every stage of development to allow for necessary adjustments to be made. These future research initiatives should involve users and health care professionals in every stage, from app design to implementation in clinical practice. This will allow them to better meet the needs of the various stakeholders through fostering their engagement.

Throughout this paper, it was also possible to highlight several elements of acceptability and feasibility for calorie-counting apps, with more general information for app structure and content. While this was partly due to the nature of our scoping review, which was to provide an overview, we still noticed that the literature was somewhat limited when it came to detailed explanations of features and functionalities, especially for commercially available apps. Therefore, future research exploring specific features and functionalities of apps and their relationship with acceptance among adults living with chronic conditions is needed.

### Conclusion

Calorie-counting apps can support management of obesity and related chronic diseases provided they reduce logging friction, personalize guidance, integrate with clinical workflows, and leverage comprehensive, culturally inclusive food databases. Persistent barriers, technical instability, incomplete databases, and the time cost of manual entry contribute to erosion of adherence over time. Future development should emphasize automation (barcode- and image-based capture, offline use), privacy-preserving clinician data sharing, and simplified monitoring paradigms that maintain engagement. Rigorous evaluations are needed to verify automated calorie estimation, benchmark database quality, and assess equity and accessibility.

## Data Availability

All data files are available from the corresponding author upon reasonable request.

## Appendix

Multimedia Appendix 1 Metadata of the extraction form used to gather the data from the 68 articles included in the scoping review.

Multimedia Appendix 2 PRISMA-ScR checklist.

## Abbreviations

mHealth: mobile health
PRISMA-ScR: Preferred Reporting Items for Systematic reviews and Meta-Analyses extension for Scoping Reviews

## References

[1] NCD Risk Factor Collaboration (NCD-RisC) (2016). Trends in adult body-mass index in 200 countries from 1975 to 2014: a pooled analysis of 1698 population-based measurement studies with 19·2 million participants. Lancet.

[2] Twells LK, Janssen I, Kuk JL (2020). Canadian adult obesity clinical practice guidelines. Obesity Canada.

[3] (2024). Population health. New Brunswick Health Council.

[4] Jiang S, Lu W, Zong X, Ruan H, Liu Y (2016). Obesity and hypertension. Exp Ther Med.

[5] Koliaki C, Liatis S, Kokkinos A (2019). Obesity and cardiovascular disease: revisiting an old relationship. Metabolism.

[6] Verma S, Hussain ME (2017). Obesity and diabetes: an update. Diabetes Metab Syndr.

[7] (2024). Obesity and overweight. World Health Organization.

[8] Wyatt HR (2013). Update on treatment strategies for obesity. J Clin Endocrinol Metab.

[9] Raber M, Liao Y, Rara A, Schembre SM, Krause KJ, Strong L, Daniel-MacDougall C, Basen-Engquist K (2021). A systematic review of the use of dietary self-monitoring in behavioural weight loss interventions: delivery, intensity and effectiveness. Public Health Nutr.

[10] (2024). Mobile fact sheet. Pew Research Center.

[11] Gareev I, Gallyametdinov A, Beylerli O, Valitov E, Alyshov A, Pavlov V, Izmailov A, Zhao S (2021). The opportunities and challenges of telemedicine during COVID-19 pandemic. Front Biosci (Elite Ed).

[12] Lieffers JRL, Hanning RM (2012). Dietary assessment and self-monitoring with nutrition applications for mobile devices. Can J Diet Pract Res.

[13] Pagoto S, Xu R, Bullard T, Foster GD, Bannor R, Arcangel K, DiVito J, Schroeder M, Cardel MI (2023). An evaluation of a personalized multicomponent commercial digital weight management program: single-arm behavioral trial. J Med Internet Res.

[14] Valinskas S, Nakrys M, Aleknavičius K, Jonusas J, Lileikienė A (2023). User engagement and weight loss facilitated by a mobile app: retrospective review of medical records. JMIR Form Res.

[15] Flores Mateo G, Granado-Font E, Ferré-Grau C, Montaña-Carreras X (2015). Mobile phone apps to promote weight loss and increase physical activity: a systematic review and meta-analysis. J Med Internet Res.

[16] Metzendorf M, Wieland LS, Richter B (2024). Mobile health (m-health) smartphone interventions for adolescents and adults with overweight or obesity. Cochrane Database Syst Rev.

[17] Yu Z, Sealey-Potts C, Rodriguez J (2015). Dietary self-monitoring in weight management: current evidence on efficacy and adherence. J Acad Nutr Diet.

[18] Arksey H, O'Malley L (2005). Scoping studies: towards a methodological framework. Int J Soc Res.

[19] Levac D, Colquhoun H, O'Brien KK (2010). Scoping studies: advancing the methodology. Implement Sci.

[20] Tricco AC, Lillie E, Zarin W, O'Brien KK, Colquhoun H, Levac D, Moher D, Peters MD, Horsley T, Weeks L, Hempel S, Akl EA, Chang C, McGowan J, Stewart L, Hartling L, Aldcroft A, Wilson MG, Garritty C, Lewin S, Godfrey CM, Macdonald MT, Langlois EV, Soares-Weiser K, Moriarty J, Clifford T, Tunçalp Ö, Straus SE (2018). PRISMA extension for scoping reviews (PRISMA-ScR): checklist and explanation. Ann Intern Med.

[21] Your personal research assistant. Zotero.

[22] Alturki R, Gay V (2019). The development of an arabic weight-loss app akser waznk: qualitative results. JMIR Form Res.

[23] Ferrara G, Kim J, Lin S, Hua J, Seto E (2019). A focused review of smartphone diet-tracking apps: usability, functionality, coherence with behavior change theory, and comparative validity of nutrient intake and energy estimates. JMIR Mhealth Uhealth.

[24] Gioia S, Vlasac IM, Babazadeh D, Fryou NL, Do E, Love J, Robbins R, Dashti HS, Lane JM (2023). Mobile apps for dietary and food timing assessment: evaluation for use in clinical research. JMIR Form Res.

[25] Kondo M, Okitsu T, Waki K, Yamauchi T, Nangaku M, Ohe K (2022). Effect of information and communication technology-based self-management system dialbeticslite on treating abdominal obesity in the specific health guidance in Japan: randomized controlled trial. JMIR Form Res.

[26] Tahir GA, Loo CK (2021). A comprehensive survey of image-based food recognition and volume estimation methods for dietary assessment. Healthcare (Basel).

[27] Kim Y, Lee H, Seo JM (2022). Integrated diabetes self-management program using smartphone application: a randomized controlled trial. West J Nurs Res.

[28] Chen J, Allman-Farinelli M (2019). Impact of training and integration of apps into dietetic practice on dietitians' self-efficacy with using mobile health apps and patient satisfaction. JMIR Mhealth Uhealth.

[29] Agustina R, Febriyanti E, Putri M, Martineta M, Hardiany NS, Mustikawati DE, Hanifa H, Shankar AH (2022). Development and preliminary validity of an Indonesian mobile application for a balanced and sustainable diet for obesity management. BMC Public Health.

[30] Pellegrini CA, Conroy DE, Phillips SM, Pfammatter AF, McFadden H, Spring B (2018). Daily and seasonal influences on dietary self-monitoring using a smartphone application. J Nutr Educ Behav.

[31] Burke LE, Sereika SM, Bizhanova Z, Parmanto B, Kariuki J, Cheng J, Beatrice B, Cedillo M, Pulantara IW, Wang Y, Loar I, Conroy MB (2022). The effect of tailored, daily, smartphone feedback to lifestyle self-monitoring on weight loss at 12 months: the smarter randomized clinical trial. J Med Internet Res.

[32] Raju VB, Sazonov E (2022). FOODCAM: A novel structured light-stereo imaging system for food portion size estimation. Sensors (Basel).

[33] Lemacks JL, Adams K, Lovetere A (2019). Dietary intake reporting accuracy of the bridge2u mobile application food log compared to control meal and dietary recall methods. Nutrients.

[34] Aizawa K, Maruyama Y, Li H, Morikawa C (2013). Food balance estimation by using personal dietary tendencies in a multimedia food log. IEEE Trans. Multimedia.

[35] Chaudhry BM (2019). Food for thought. Mhealth.

[36] Anthimopoulos M, Dehais J, Shevchik S, Ransford BH, Duke D, Diem P, Mougiakakou S (2015). Computer vision-based carbohydrate estimation for type 1 patients with diabetes using smartphones. J Diabetes Sci Technol.

[37] Olfert MD, Barr ML, Hagedorn RL, Long DM, Haggerty TS, Weimer M, Golden J, Maurer MA, Cochran JD, Hendershot T, Whanger SL, Mason JD, Hodder SL (2019). Feasibility of a mHealth approach to nutrition counseling in an appalachian state. J Pers Med.

[38] (2015). iDAT app (version 2.2.0). Health Promotion Board.

[39] Myers A, Johnston N, Rathod V, Korattikara A, Gorban A, Silberman N, Guadarrama S, Papandreou G, Huang J, Murphy K (2015). Im2Calories: towards an automated mobile vision food diary.

[40] Zahedani AD, McLaughlin T, Veluvali A, Aghaeepour N, Hosseinian A, Agarwal S, Ruan J, Tripathi S, Woodward M, Hashemi N, Snyder M (2023). Digital health application integrating wearable data and behavioral patterns improves metabolic health. NPJ Digit Med.

[41] Moyen A, Rappaport AI, Fleurent-Grégoire C, Tessier A, Brazeau A, Chevalier S (2022). Relative validation of an artificial intelligence-enhanced, image-assisted mobile app for dietary assessment in adults: randomized crossover study. J Med Internet Res.

[42] Schusterbauer V, Feitek D, Kastner P, Toplak H (2018). Two-stage evaluation of a telehealth nutrition management service in support of diabesity therapy. Stud Health Technol Inform.

[43] Oh SW, Kim K, Kim SS, Park SK, Park S (2022). Effect of an integrative mobile health intervention in patients with hypertension and diabetes: crossover study. JMIR Mhealth Uhealth.

[44] Burke LE, Zheng Y, Ma Q, Mancino J, Loar I, Music E, Styn M, Ewing L, French B, Sieworek D, Smailagic A, Sereika SM (2017). The SMARTER pilot study: testing feasibility of real-time feedback for dietary self-monitoring. Prev Med Rep.

[45] Beijbom O, Joshi N, Morris D, Saponas S, Khullar S (2015). Menu-menu-match: restaurant-specific food logging from images.

[46] Ahmad Z, Kerr DA, Bosch M, Boushey CJ, Delp EJ, Khanna N, Zhu F (2016). A mobile food record for integrated dietary assessment. MADiMa16 (2016).

[47] Chui T, Tan J, Li Y, Raynor HA (2020). Validating an automated image identification process of a passive image-assisted dietary assessment method: proof of concept. Public Health Nutr.

[48] Tosi M, Radice D, Carioni G, Vecchiati T, Fiori F, Parpinel M, Gnagnarella P (2021). Accuracy of applications to monitor food intake: evaluation by comparison with 3-d food diary. Nutrition.

[49] Carter MC, Burley VJ, Nykjaer C, Cade JE (2013). Adherence to a smartphone application for weight loss compared to website and paper diary: pilot randomized controlled trial. J Med Internet Res.

[50] Fu HNC, Rizvi RF, Wyman JF, Adam TJ (2020). Usability evaluation of four top-rated commercially available diabetes apps for adults with type 2 diabetes. Comput Inform Nurs.

[51] Lim SL, Tay MHJ, Ong KW, Johal J, Yap QV, Chan YH, Yeo GKN, Khoo CM, Yaxley A (2022). Association between mobile health app engagement and weight loss and glycemic control in adults with type 2 diabetes and prediabetes (D'LITE Study): prospective cohort study. JMIR Diabetes.

[52] Jin T, Kang G, Song S, Lee H, Chen Y, Kim S, Shin M, Park YH, Lee JE (2023). The effects of dietary self-monitoring intervention on anthropometric and metabolic changes via a mobile application or paper-based diary: a randomized trial. Nutr Res Pract.

[53] Kay MC, Miller HN, Askew S, Spaulding EM, Chisholm M, Christy J, Yang Q, Steinberg DM (2022). Patterns of engagement with an application-based dietary self-monitoring tool within a randomized controlled feasibility trial. AJPM Focus.

[54] Zhou J, Bell D, Nusrat S, Hingle M, Surdeanu M, Kobourov S (2018). Calorie estimation from pictures of food: crowdsourcing study. Interact J Med Res.

[55] Gonzalez-Ramirez M, Sanchez-Carrera R, Cejudo-Lopez A, Lozano-Navarrete M, Salamero Sánchez-Gabriel E, Torres-Bengoa MA, Segura-Balbuena M, Sanchez-Cordero MJ, Barroso-Vazquez M, Perez-Barba FJ, Troncoso AM, Garcia-Parrilla MC, Cerezo AB (2022). Short-term pilot study to evaluate the impact of salbi educa nutrition app in macronutrients intake and adherence to the mediterranean diet: randomized controlled trial. Nutrients.

[56] Zhang W, Yu Q, Siddiquie B, Divakaran A, Sawhney H (2015). "Snap-n-Eat": food recognition and nutrition estimation on a smartphone. J Diabetes Sci Technol.

[57] Bardus M, Ali A, Demachkieh F, Hamadeh G (2019). Assessing the quality of mobile phone apps for weight management: user-centered study with employees from a lebanese university. JMIR Mhealth Uhealth.

[58] Riddell M, Li Z, Gal R, Calhoun P, Jacobs P, Clements M, Martin C, Doyle Iii FJ, Patton S, Castle J, Gillingham M, Beck R, Rickels M, T1DEXI Study Group (2023). Examining the acute glycemic effects of different types of structured exercise sessions in type 1 diabetes in a real-world setting: the type 1 diabetes and exercise initiative (T1DEXI). Diabetes Care.

[59] O'Neil PM, Miller-Kovach K, Tuerk PW, Becker LE, Wadden TA, Fujioka K, Hollander PL, Kushner RF, Timothy Garvey W, Rubino DM, Malcolm RJ, Weiss D, Raum WJ, Salyer JL, Hermayer KL, Rost SL, Veliko JL, Sora ND (2016). Randomized controlled trial of a nationally available weight control program tailored for adults with type 2 diabetes. Obesity (Silver Spring).

[60] Puigdomènech E, Robles N, Balfegó M, Cuatrecasas G, Zamora A, Saigí-Rubió F, Paluzié G, Moharra M, Carrion C (2022). Codesign and feasibility testing of a tool to evaluate overweight and obesity apps. Int J Environ Res Public Health.

[61] Ifejika NL, Bhadane M, Cai CC, Noser EA, Grotta JC, Savitz SI (2020). Use of a smartphone-based mobile app for weight management in obese minority stroke survivors: pilot randomized controlled trial with open blinded end point. JMIR Mhealth Uhealth.

[62] Ben Neriah D, Geliebter A (2019). Weight loss following use of a smartphone food photo feature: retrospective cohort study. JMIR Mhealth Uhealth.

[63] Hahn SL, Kaciroti N, Eisenberg D, Weeks HM, Bauer KW, Sonneville KR (2021). Introducing dietary self-monitoring to undergraduate women via a calorie counting app has no effect on mental health or health behaviors: results from a randomized controlled trial. J Acad Nutr Diet.

[64] Baum Martinez I, Peters B, Schwarz J, Schuppelius B, Steckhan N, Koppold-Liebscher DA, Michalsen A, Pivovarova-Ramich O (2022). Validation of a smartphone application for the assessment of dietary compliance in an intermittent fasting trial. Nutrients.

[65] Chew H, Lim S, Kim G, Kayambu G, So B, Shabbir A, Gao Y (2023). Essential elements of weight loss apps for a multi-ethnic population with high BMI: a qualitative study with practical recommendations. Transl Behav Med.

[66] Vasiloglou MF, Christodoulidis S, Reber E, Stathopoulou T, Lu Y, Stanga Z, Mougiakakou S (2021). Perspectives and preferences of adult smartphone users regarding nutrition and diet apps: web-based survey study. JMIR Mhealth Uhealth.

[67] Patel ML, Hopkins CM, Brooks TL, Bennett GG (2019). Comparing self-monitoring strategies for weight loss in a smartphone app: randomized controlled trial. JMIR Mhealth Uhealth.

[68] Wharton CM, Johnston CS, Cunningham BK, Sterner D (2014). Dietary self-monitoring, but not dietary quality, improves with use of smartphone app technology in an 8-week weight loss trial. J Nutr Educ Behav.

[69] Kwon BC, VanDam C, Chiuve SE, Choi HW, Entler P, Tan P, Huh-Yoo J (2020). Improving heart disease risk through quality-focused diet logging: pre-post study of a diet quality tracking app. JMIR Mhealth Uhealth.

[70] Kim HH, Kim Y, Michaelides A, Park YR (2022). Weight loss trajectories and related factors in a 16-week mobile obesity intervention program: retrospective observational study. J Med Internet Res.

[71] Eisenhauer CM, Brito F, Kupzyk K, Yoder A, Almeida F, Beller RJ, Miller J, Hageman PA (2021). Mobile health assisted self-monitoring is acceptable for supporting weight loss in rural men: a pragmatic randomized controlled feasibility trial. BMC Public Health.

[72] Gonzalez-Ramirez M, Cejudo-Lopez A, Lozano-Navarrete M, Salamero Sánchez-Gabriel E, Torres-Bengoa MA, Segura-Balbuena M, Sanchez-Cordero MJ, Barroso-Vazquez M, Perez-Barba FJ, Troncoso AM, Garcia-Parrilla MC, Cerezo AB (2022). SAlBi educa (Tailored Nutrition App for Improving Dietary Habits): initial evaluation of usability. Front Nutr.

[73] Lee J, Bae S, Park D, Kim Y, Park J (2020). The effectiveness of a monetary reimbursement model for weight reduction via a smartphone application: a preliminary retrospective study. Sci Rep.

[74] Chew HSJ, Achananuparp P, Dalakoti M, Chew NWS, Chin YH, Gao Y, So BYJ, Shabbir A, Peng LE, Ngiam KY (2024). Public acceptance of using artificial intelligence-assisted weight management apps in high-income southeast Asian adults with overweight and obesity: a cross-sectional study. Front Nutr.

[75] Zheng Y, Weinger K, Greenberg J, Burke LE, Sereika SM, Patience N, Gregas MC, Li Z, Qi C, Yamasaki J, Munshi MN (2020). Actual use of multiple health monitors among older adults with diabetes: pilot study. JMIR Aging.

[76] Pagoto S, Tulu B, Waring ME, Goetz J, Bibeau J, Divito J, Groshon L, Schroeder M (2021). Slip buddy app for weight management: randomized feasibility trial of a dietary lapse tracking app. JMIR Mhealth Uhealth.

[77] Pagoto S, Xu R, Bullard T, Foster GD, Bannor R, Arcangel K, DiVito J, Schroeder M, Cardel MI (2023). An evaluation of a personalized multicomponent commercial digital weight management program: single-arm behavioral trial. J Med Internet Res.

[78] Goh G, Tan NC, Malhotra R, Padmanabhan U, Barbier S, Allen JC, Østbye T (2015). Short-term trajectories of use of a caloric-monitoring mobile phone app among patients with type 2 diabetes mellitus in a primary care setting. J Med Internet Res.

[79] Laing BY, Mangione CM, Tseng C, Leng M, Vaisberg E, Mahida M, Bholat M, Glazier E, Morisky DE, Bell DS (2014). Effectiveness of a smartphone application for weight loss compared with usual care in overweight primary care patients: a randomized, controlled trial. Ann Intern Med.

[80] Alshathri DM, Alhumaimeedy AS, Al-Hudhud G, Alsaleh A, Al-Musharaf S, Aljuraiban GS (2020). Weight management apps in Saudi Arabia: evaluation of features and quality. JMIR Mhealth Uhealth.

[81] Rowland S, Ramos AK, Trinidad N, Quintero S, Johnson Beller R, Struwe L, Pozehl B (2022). Feasibility, usability and acceptability of a mhealth intervention to reduce cardiovascular risk in rural hispanic adults: descriptive study. JMIR Form Res.

[82] Carter MC, Burley VJ, Cade JE (2017). Weight loss associated with different patterns of self-monitoring using the mobile phone app my meal mate. JMIR Mhealth Uhealth.

[83] Garizábalo-Dávila CM, Rodríguez-Acelas AL, Cañon-Montañez W (2023). Usefulness of acceptability and feasibility assessment in studies of nursing interventions. Invest Educ Enferm.

[84] Patel ML, Cleare AE, Smith CM, Rosas LG, King AC (2022). Detailed versus simplified dietary self-monitoring in a digital weight loss intervention among racial and ethnic minority adults: fully remote, randomized pilot study. JMIR Form Res.

[85] Banerjee P, Mendu VVR, Korrapati D, Gavaravarapu SM (2020). Calorie counting smart phone apps: effectiveness in nutritional awareness, lifestyle modification and weight management among young Indian adults. Health Informatics J.

[86] (2024). Extender app (Version 5.7.1. Food Database GmbH (FDDB).

[87] Butryn ML, Godfrey KM, Martinelli MK, Roberts SR, Forman EM, Zhang F (2020). Digital self-monitoring: does adherence or association with outcomes differ by self-monitoring target?. Obes Sci Pract.

[88] Li S, Zhou Y, Tang Y, Ma H, Zhang Y, Wang A, Tang X, Pei R, Piao M (2025). Behavior change resources used in mobile app-based interventions addressing weight, behavioral, and metabolic outcomes in adults with overweight and obesity: systematic review and meta-analysis of randomized controlled trials. JMIR Mhealth Uhealth.

[89] Lehmann M, Jones L, Schirmann F (2024). App engagement as a predictor of weight loss in blended-care interventions: retrospective observational study using large-scale real-world data. J Med Internet Res.

