# Supplementary material for: Calorie-Counting Apps for Monitoring and Managing Calorie Intake in Adults Living With Weight-Related Chronic Diseases: Decade-Long Scoping Review (2013-2024)
Source: JMIR Mhealth Uhealth. 2026 Apr 1;14:e64139. doi: 10.2196/64139 (PMC13085986; doi:10.2196/64139)
Supplement: Multimedia Appendix 1 [file mhealth_v14i1e64139_app1.docx]

This is a Multimedia Appendix to the report entitled “Calorie Counting Apps for Monitoring and Managing Calorie Intake in Adults with Weight-Related Chronic Diseases: A Decade-long Scoping Review (2014-2024).” This report was produced during a public health rotation by four medical students, Kaylee Rose Dugas, Abdelatif Guerroudj, Marie-Andrée Giroux, and Jazna Léger, under the supervision of the principal investigator, Prof. Dr. Jalila Jbilou, and in collaboration with computer science researchers Dr. Asal Rouhafzay and Prof. Dr. Ghazal Rouhafzay.

**Table S1.** Metadata of the extraction form used to gather the data from the 68 articles included in the scoping review.

| **COLUMN TITLE** | **DESCRIPTION (WHEN COLUMN TITLE IS NOT SELF-EXPLANATORY)** |
| --- | --- |
| PMID | Article unique ID |
| Title |  |
| Author(s) |  |
| Year |  |
| Specific notes concerning article | If presence of relevant information about the article that doesn't fit in other columns. |
| Student | Name of the student who have extracted the data |
| Objectives | Objectives or the article |
| Geographical location of study (country) |  |
| Research protocol Type of study (RCT, etc...) |  |
| Theoretical framework | Theoretical, Conceptual, Framework, Model |
| Characteristics of chronic illness in study population (insert table, inclusion exclusion criteria, adult, elderly, ethnic group, type of chronic disease, community dwelling vs in hospital/ clinical) | Type of chronic illness (e.g. diabetes, obesity, overweight, CV diseases, etc.) and associated characteristics (mean and interval of BMI values, etc.), community or hospitalized, race if specified, etc. |
| Measurements: (surveys with questions used, any scores used? How was adherence measured?( |  |
| Names and objectives of (calorie tracking) apps studied ( if no specific app mentionned, specify it) |  |
| How is the information saved in the app? | Information related to whether users needed to create an account. |
| How is information logged in order to get the calories counted? (Manually? Picture based? Barcode scanning?) |  |
| How does the app allow monitoring of data by HCP? (and platform used if applicable) |  |
| What are the features for goal setting (ex: I want to attain a certain weight, I want to eat this amount of calories)? |  |
| How does the app interact with the user (feedback/reminders/health prompts)? |  |
| Are there other pertinent key design features used (PREMIUM feature?, use of color, interactive prompts, layout of the app (clean design vs cluttered))? |  |

**ACCEPTABILITY**

| **COLUMN TITLE** | **DESCRIPTION (WHEN COLUMN TITLE IS NOT SELF-EXPLANATORY)** |
| --- | --- |
| Patient : How user friendly is the app? (System user scoring SUS, perception of participants in terms of ease of use, time to learn how to use app etc...). If so, why is the app considered user friendly? |  |
| HCP : How user friendly is the app? (System user scoring SUS, perception of participants in terms of ease of use, time to learn how to use app etc...). If so, why is the app considered user friendly? |  |
| How usable (USEFUL) is the app to the user? (their perception) |  |
| How usable (USEFUL) is the app to the Health care professionnals? (their perception) |  |
| What are the barriers limiting access of this app (pay to use, premium subscription, no features for disabled people, not available on certain markets (apple/ samsung), not available on every type of device, language, e-literacy, app not working good bugs crashes,) ACCORDING TO USER |  |
| What are the barriers limiting access of this app (pay to use, premium subscription, no features for disabled people, not available on certain markets (apple/ samsung), not available on every type of device, language, e-literacy, app not working good bugs crashes,) HCP if applicable) |  |
| Acceptability - others | Other information related to acceptability that does not fit in other columns. |

**FEASIBILITY**

| **COLUMN TITLE** | **DESCRIPTION (WHEN COLUMN TITLE IS NOT SELF-EXPLANATORY)** |
| --- | --- |
| Does the app provide evidence based informations and recommandations? | This column also includes whether the app is based on expert knowledge |
| Does the app provide accurate calorie counting (for example, is there a comprehensive food data base?) |  |
| Is there any other limitations for implementing this app in a clinical setting? (lack of data privacy, lack of time to implement in clinical setting, lack of etc, cost effectiveness/affordability, frequently updated, too demanding/technological fatigue and any other features that limit usability...) ACCORDING TO USER |  |
| Is there any other limitations for implementing this app in a clinical setting? (lack of data privacy, lack of time to implement in clinical setting, lack of etc, cost effectiveness/affordability, frequently updated, too demanding/technological fatigue and any other features that limit usability...) ACCORDING TO HCP |  |
| Feasbility - others | Other information related to feasibility that does not fit in other columns. |
